# Protective Effect of Diphlorethohydroxycarmalol Isolated from *Ishige okamurae* Against Particulate Matter-Induced Skin Damage by Regulation of NF-κB, AP-1, and MAPKs Signaling Pathways In Vitro in Human Dermal Fibroblasts

**DOI:** 10.3390/molecules25051055

**Published:** 2020-02-26

**Authors:** Lei Wang, Hyun Soo Kim, Jun-Geon Je, Jae Young Oh, Young-Sang Kim, Seon-Heui Cha, You-Jin Jeon

**Affiliations:** 1 Department of Marine Life Sciences, Jeju National University, Jeju Self-Governing Province 63243, Korea; comeonleiwang@163.com (L.W.); wpwnsrjs@naver.com (J.-G.J.); ojy0724@naver.com (J.Y.O.); medieval032@naver.com (Y.-S.K.); 2 Marine Science Institute, Jeju National University, Jeju Self-Governing Province 63333, Korea; 3 Department of Applied Research, National Marine Biodiversity Institute of Korea, 75, Jangsan-ro 101-gil, Janghang-eup, Seocheon 33675, Korea; gustn783@mabik.re.kr; 4 Department of Marine Bioindustry, Hanseo University, Chungcheognam-do 32158, Korea

**Keywords:** particulate matter, skin damage, diphlorethohydroxycarmalol, *Ishige okamurae*, algal polyphenol, human dermal fibroblast

## Abstract

Particulate matters (PM), the main contributor to air pollution, have become a serious issue that threatens human’s health. Skin is the largest organ in humans, as well as the primary organ exposed to PM. Overexposure of PM induces skin damage. Diphlorethohydroxycarmalol (DPHC), an algal polyphenol with the potential of skin protection, has been isolated from the edible brown seaweed *Ishige okamurae*. The purpose of the present study is to investigate the protective effect of DPHC against PM (ERM-CZ100)-induced skin damage in human dermal fibroblasts (HDF) cells. The results indicated that DPHC significantly and dose-dependently reduced intracellular reactive oxygen species generation in HDF cells. In addition, DPHC significantly induced collagen synthesis and inhibited collagenase activity in ERM-CZ100-stimulated HDF cells. Further study demonstrated that DPHC remarkably reduced the expression of human matrix metalloproteinases through regulation of nuclear factor kappa B, activator protein 1, and mitogen-activated protein kinases signaling pathways in ERM-CZ100-stimulated HDF cells. This study suggested that DPHC is a potential candidate to protect skins against PM-induced damage, and it could be used as an ingredient in pharmaceutical and cosmeceutical industries.

## 1. Introduction

Particulate matters (PM) are one of the components causing ambient air pollution. In general, PM were produced by human activity, such as chemical production and fossil fuel combustion [1,2]. PM are related to various diseases, including inflammations such as skin inflammation and lung inflammation, kidney diseases such as chronic kidney disease and end-stage renal disease, metabolic syndromes such as diabetes and obesity, as well as skin aging such as wrinkle formation [3,4,5,6,7,8,9,10,11]. Skin is the largest organ in humans and the primary organ exposed to PM. A long-term exposure to PM causes many negative effects on the skin, such as oxidative stress, inflammatory response, atopic dermatitis, aging, and skin carcinoma [12,13,14,15,16]. In recent years, the related studies have been done to investigate the relationship between PM and skin health, as well as to discover the agents that could protect or improve PM-induced skin damages.

Marine-derived natural components, such as phenolic compounds, peptides, polysaccharides, sterols, pigments, and fatty acids from marine animals, microorganisms, and seaweeds, possess various bioactivities, including anti-malarial, antioxidant, anti-bacterial, anti-cancer, anti-hypertensive, anti-inflammatory, anti-obesity, wound healing, and anti-diabetes activities [17,18,19,20,21,22,23,24,25]. Ko et al. (2016) purified peptides from Olive flounder and investigated their anti-hypertensive activity in in vitro and in vivo models [26]. Wang et al. (2019) isolated sulfated polysaccharides from the brown seaweed, *Sargassum fulvellum,* and evaluated the in vitro and in vivo antioxidant activities of the sulfated polysaccharides [27]. Pang et al. (2018) isolated perylenequinone derivatives from marine sponge-derived fungus and investigated the anti-cancer activities of these compounds [28].

**Figure 1:**
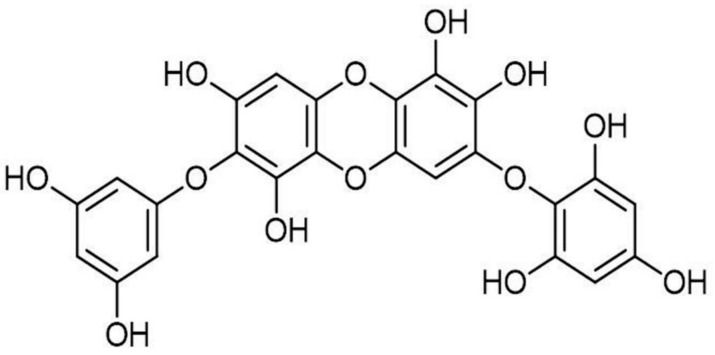
The molecular structure of diphlorethohydroxycarmalol (DPHC).

*Ishige okamurae* is one of the most popular edible seaweeds in Korea. Many bioactive compounds have been isolated and identified from *I. okamurae*, such as polysaccharides, pigments, and phenolic compounds [29,30,31,32]. Besides, the bioactivities of these compounds have been evaluated [30,33,34]. Diphlorethohydroxycarmalol (DPHC, Figure 1), a phenolic compound derived from *I. okamurae*, which possesses the potential for skin protection [34,35,36]. Previous studies suggested that DPHC could effectively protect skins against epidermic damages induced by UVB irradiation and PM in human keratinocytes (HaCaT cells) [36,37]. In addition, the protective effect of DPHC on UVB-induced dermic damages in human dermal fibroblasts (HDF cells) has been investigated [34]. However, the effect of DPHC on PM-induced dermic damage has not been investigated so far. In the present study, therefore, the effect of DPHC on PM-induced dermic damages in in vitro human dermal fibroblasts has been examined.

## 2. Results

### 2.1. DPHC Improves PM-Induced Oxidative Damage in HDF Cells

**Figure 2:**
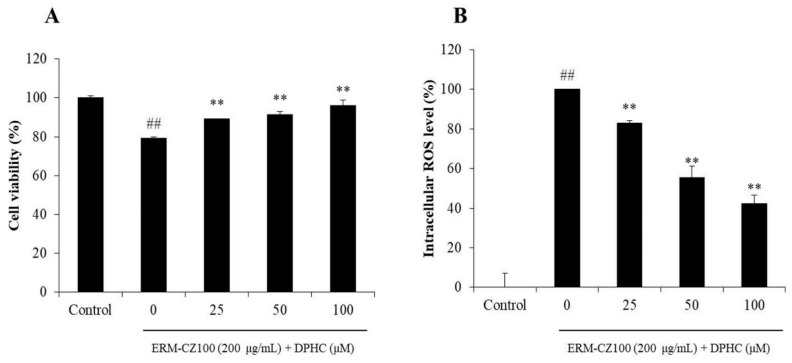
Protective effect of DPHC against ERM-CZ100-induced human dermal fibroblasts (HDF cells) damage. (**A**) Protective effect of DPHC against ERM-CZ100-induced cell death and (**B**) intracellular ROS scavenging effect of DPHC in ERM-CZ100-stimulated HDF cells. Cell viability was measured by MTT assay, and intracellular ROS level was measured by DCF-DA assay. The data are expressed as the mean ± SE (*n* = 3). ** *p* < 0.01 as compared to the particulate matters (PM)-treated group and ^##^ *p* < 0.01 as compared to the control group.

It has been reported that PM (ERM-CZ-100) caused oxidative damage on HDF cells [8]. In the present study, the effect of DPHC on cell death and intracellular reactive oxygen species (ROS) generation in PM-stimulated HDF cells was measured. As shown in Figure 2A, the viability of the cells treated with ERM-CZ-100 was significantly decreased. However, the viabilities of the cells treated with different concentrations of DPHC were remarkably and dose-dependently increased. In addition, ERM-CZ-100 significantly induced intracellular ROS generation in HDF cells, but DPHC remarkably reduced intracellular ROS levels in a dose-dependent manner (Figure 2B). These results indicated that DPHC effectively protected HDF cells against PM-induced cell death by reducing intracellular ROS level.

### 2.2. DPHC Protects Collagen Synthesis and Inhibits Intracellular Collagenase Activity in PM-Stimulated HDF Cells

**Figure 3:**
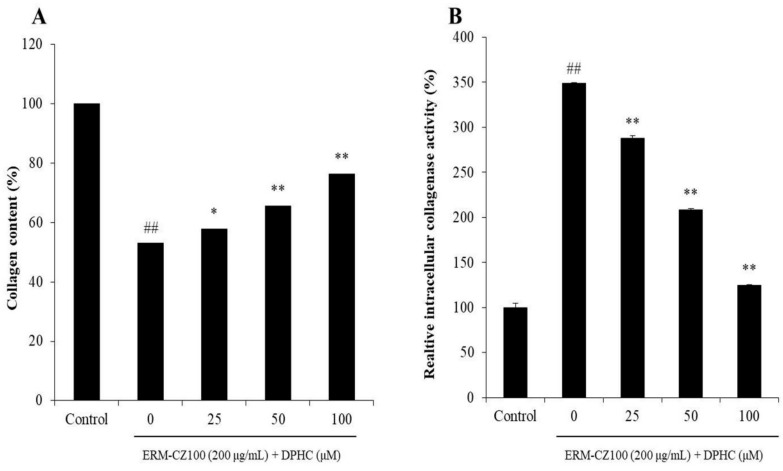
DPHC protected collagen synthesis and inhibited intracellular collagenase activity in ERM-CZ100-stimulated HDF cells. (**A**) The effect of DPHC on collagen synthesis in ERM-CZ100-stimulated HDF cells, and (**B**) the effect of DPHC on relative collagenase activity in ERM-CZ100-stimulated HDF cells. The data were expressed as the mean ± SE (*n* = 3). * *p* < 0.05, ** *p* < 0.01 as compared to the ERM-CZ100-treated group, and ^##^ *p* < 0.01 as compared to the control group.

**Figure 4:**
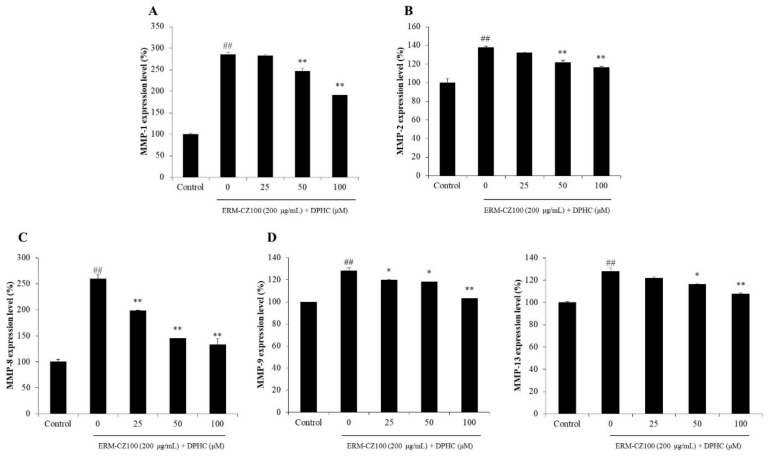
DPHC reduced matrix metalloproteinases (MMPs) expression in ERM-CZ100-stimulated HDF cells. (**A**) MMP-1 expression level in ERM-CZ100-stimulated HDF cells, (**B**) MMP-2 expression level in ERM-CZ100-stimulated HDF cells, (**C**) MMP-8 expression level in ERM-CZ100-stimulated HDF cells, (**D**) MMP-9 expression level in ERM-CZ100-stimulated HDF cells, and (**E**) MMP-13 expression level in ERM-CZ100-stimulated HDF cells. The data were expressed as the mean ± SE (*n* = 3). * *p* < 0.05 and ** *p* < 0.01 as compared to the ERM-CZ100-treated group, and ^#^ *p* < 0.05 and ^##^ *p* < 0.01 as compared to the control group.

The collagen synthesis levels and intracellular collagenase activities of ERM-CZ-100-stimulated HDF cells with or without DPHC were determined. As shown in Figure 3A, the collagen synthesis level in ERM-CZ-100-stimulated HDF cells was significantly decreased but remarkably increased by DPHC in a dose-dependent manner. In addition, the intracellular collagenase activity in ERM-CZ-100-stimulated HDF cells was significantly increased but remarkably and dose-dependently decreased with the DPHC treatments (Figure 4B). These results indicated that DPHC not only improved collagen synthesis but also inhibited intracellular collagenase activity in ERM-CZ-100-stimulated HDF cells.

### 2.3. DPHC Reduces Matrix Metalloproteinases (MMPs) Expression in PM-Stimulated HDF Cells

MMPs expression levels in ERM-CZ-100-stimulated HDF cells were measured by ELISA kits, and the results were indicated in Figure 4. ERM-CZ-100 significantly stimulated MMPs expression and DPHC effectively inhibited the expression of MMPs in a dose-dependent manner, especially MMP-1 and MMP-8. These results indicated that DPHC could effectively inhibit the expression of MMPs expression stimulated by ERM-CZ-100 in HDF cells.

### 2.4. DPHC Inhibits Nuclear Factor Kappa B (NF-κB) Activation, Reduces Activator Protein (AP-1) Phosphorylation, and Suppresses Mitogen-Activated Protein Kinases (MAPKs) Activation in PM-Stimulated HDF Cells

**Figure 5:**
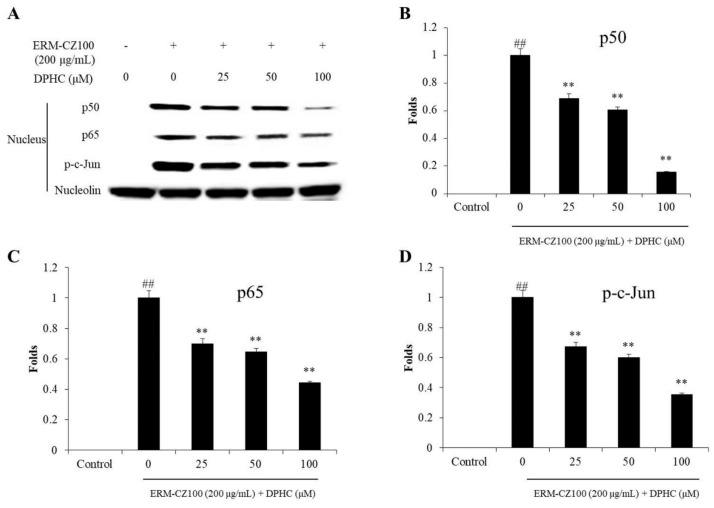
DPHC inhibited NF-κB activation and AP-1 phosphorylation in ERM-CZ100-stimulated HDF cells. (**A**) The nuclear NF-κB-related proteins (p65 and p50) and phosphorylated AP-1 (p-c-Jun) levels in ERM-CZ100-stimulated HDF cells, and relative amounts of NF-κB p50 (**B**), NF-κB p65 (**C**), and p-c-Jun (**D**) levels in ERM-CZ100-stimulated HDF cells. The relative amounts of NF-κB p65, NF-κB p50, and p-c-Jun levels were compared with nucleolin. The data were expressed as the mean ± SE (*n* = 3). * *p* < 0.05, ** *p* < 0.01 as compared to the ERM-CZ100-treated group and ^##^ *p* < 0.01 as compared to the control group.

The effect of DPHC on NF-κB, AP-1, and MAPKs pathways was investigated. As shown in Figure 5, the nucleus p50 and p60 NF-κB subunits were increased in ERM-CZ-100-treated HDF cells. However, both subunits were decreased in the cells treated with DPHC in a dose-dependent manner (Figure 5A–C). In addition, ERM-CZ-100 significantly induced AP-1 (c-Jun) phosphorylation, and the phosphorylated AP-1 (p-c-Jun) levels were significantly and dose-dependently decreased in the cells treated with DPHC (Figure 5A,D).

**Figure 6:**
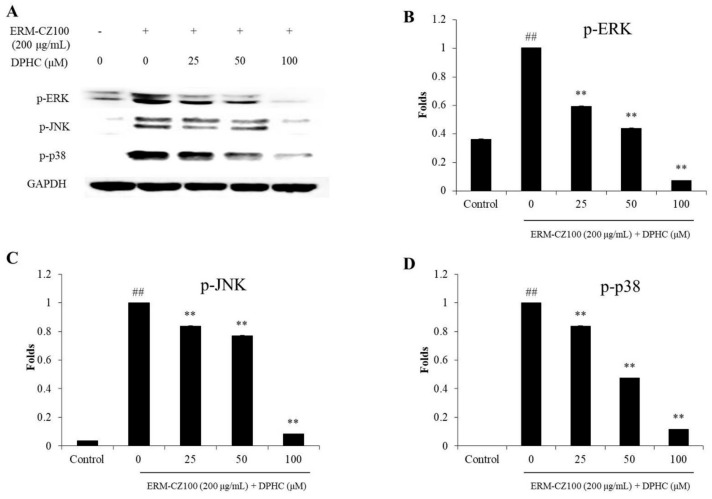
DPHC suppressed MAPKs activation in ERM-CZ100-stimulated HDF cells. (**A**) The inhibitory effects of DPHC on ERM-CZ100-stimulated MAPKs activation in HDF cells, and relative amounts of activated p-ERK (**B**), p-JNK (**C**), and p-p38 (**D**) levels in ERM-CZ100-stimulated HDF cells. The relative amounts of activated MAPKs levels were compared with GAPDH. The data were expressed as the mean ± SE (*n* = 3). * *p* < 0.05 and ** *p* < 0.01 as compared to the ERM-CZ100-treated group and ^##^ *p* < 0.01 as compared to the control group.

Furthermore, DPHC remarkably suppressed the phosphorylation of p38, ERK, and JNK MAPKs in ERM-CZ-100-stimulated HDF cells (Figure 6). These results demonstrated that DPHC significantly inhibited the activation of NF-κB, AP-1, and MAPKs pathways induced by ERM-CZ-100.

## 3. Discussion

Skin is the largest organ of the human body, as well as the primary organ which is exposed to outdoor contaminants. Exposure to PM causes epithelium damage, and endothelial dysfunction has been reported previously [8,9,38]. PM induce skin damages or diseases mainly through stimulating inflammatory response and oxidative stress [5,39]. Ryu et al. investigated that PM induced epidermic inflammatory response in human keratinocytes [39]. The results indicated that PM significantly stimulated the expression of pro-inflammatory cytokines through activating NF-κB pathway [39]. Piao et al. evaluated the effect of PM on human keratinocytes, and the results indicated that PM significantly induced oxidative stress by production of ROS, which causes lipid peroxidation, DNA damage, and protein carbonylation [9].

In the previous study, we investigated the effect of PM on HDF cells [8]. The results indicated that PM significantly induced skin dermic damage by inhibiting collagen synthesis and inducing MMPs expression in HDF cells through activating NF-κB, AP-1, and MAPKs signaling pathways [8]. Furthermore, the effect of the phenolic compound, epigallocatechin gallate (EGCG) from green tea on PM-induced dermic damage in HDF cells, has been evaluated. The results indicated that EGCG was effectively protective of HDF cells against PM-induced dermic damage displayed in improving collagen synthesis and reducing MMPs expression through inhibiting NF-κB, AP-1, and MAPKs signaling pathways [8]. These results demonstrated that PM could induce dermic damage and its possible mechanism in the activation of NF-κB, AP-1, and MAPKs signaling pathways. Besides, this study suggested that PM-induced dermic damage could be protected or suppressed by natural bioactive compounds through regulating the relative signaling pathways including NF-κB, AP-1, and MAPKs.

DPHC is one of the most abundant phenolic compounds of the edible brown alga *I. okamurae*. It possesses the potential for skin protection but no toxicity [34,35,36]. In the present study, the effect of DPHC on PM-induced dermic damage in vitro in HDF cells has been examined. As shown in Figure 2, DPHC effectively protected HDF cells against PM-induced intracellular ROS generation (Figure 2A) and cell death (Figure 2B) in a dose-dependent manner. Collagenase is the key enzyme in the degradation of collagen, which is the main structural protein in the connective tissue. The previous study indicated that PM inhibited collagen synthesis and stimulated collagenase in HDF cells [8]. Therefore, in the present study, the collagen synthesis levels and relative intracellular collagenase activity in PM-stimulated HDF cells was investigated. As shown in Figure 3A, DPHC improved collagen content to 57.82%, 65.67%, and 76.32% at the concentration of 25, 50, and 100 μM in ERM-CZ-100-treated HDF cells, respectively, compared to the cells treated with ERM-CZ-100 only (53.16%). Additionally, ERM-CZ-100 increased the relative intracellular collagenase activity in HDF cells to 348.64% compared to the control (100%) (Figure 3B). However, the relative intracellular collagenase activities of ERM-CZ-100-treated HDF cells were decreased to 287.66%, 208.53%, and 124.86% by being treated with 25, 50, and 100 μM of DPHC (Figure 3B). It indicated that DPHC not only protected collagen synthesis but also suppressed collagen degradation by inhibiting the relative intracellular collagenase activity in ERM-CZ-100-stimulated HDF cells.

MMPs are different kinds of collagenase and related to various diseases, such as cancer and skin aging [8,34]. In this study, the MMPs levels including MMP-1, 2, 3, 9, and 13, which are associated with collagen degradation and wrinkle formation in aged skin, were evaluated. As shown in Figure 4, DPHC significantly and dose-dependently inhibited MMPs expression stimulated by ERM-CZ-100, especially MMP-1 and MMP-8. Many studies suggested that the expressions of MMPs are related to NF-κB, AP-1, and MAPKs signaling pathways [8,40]. NF-κB is a complex that is generally located in the plasma of cells. However, it can be activated by various stimulators, such as chemicals, UV irradiation, and PM. The activated NF-κB subunits (p50 and p65) translated into the nucleus could induce the expression of pro-inflammatory cytokines and MMPs [34,41]. AP-1 could promote several MMPs expression, and it was activated by the phosphorylated MAPKs, which activate by ROS overproduction [34,37]. Thus, in the present study, the activated NF-κB, AP-1, and MAPKs were measured by Western blot assay. As Figure 5 shows, ERM-CZ-100 significantly increased the nucleus NF-κB p50 and p65 levels; however, DPHC remarkably and dose-dependently reduced the nucleus NF-κB p50 and p65 levels (Figure 5A–C). Besides, DPHC significantly reduced the activated AP-1 (p-c-Jun) level in a dose-dependent manner (Figure 5A and D). Furthermore, the phosphorylated MAPKs levels, including p-P38, p-ERK, and p-JNK, were significantly increased in ERM-CZ-100-treated HDF cells and remarkably decreased in DPHC-treated HDF cells in a dose-dependent manner (Figure 6). These results demonstrated that DPHC inhibited MMPs expression by regulation of NF-κB, AP-1, and MAPKs signaling pathways, which may owe to its intracellular ROS scavenging effects.

In conclusion, in the present study, the effect of DPHC on PM-induced skin dermic damage was investigated in vitro in HDF cells. The results indicated that DPHC significantly increased cell viability and reduced intracellular ROS level in ERM-CZ-100-stimulated HDF cells. In addition, DPHC effectively protected collagen synthesis and inhibited collagen degradation by inhibiting the relative intracellular collagenase in ERM-CZ-100-stimulated HDF cells. Furthermore, DPHC significantly reduced ERM-CZ-100-stimulated MMPs expression through regulating NF-κB, AP-1, and MAPKs signaling pathways. These results suggested that DPHC possesses strong effects against PM-induced dermic damage. In addition, our previous research evaluated the toxicity of DPHC in vitro in HDF cells and in vivo in zebrafish, as well as investigated the photoprotective effect of DPHC [34]. The results indicated that DPHC possesses a strong in vitro and in vivo photoprotective effect without toxicity [34]. Taken together, the previous and present data supported that DPHC has the potential to be used as an ingredient in pharmaceutical and cosmeceutical industries. However, to develop DPHC as a therapeutic agent or cosmetic to treat skin damage, the clinical study is vital in further research.

## 4. Materials and Methods

### 4.1. Chemicals and Reagents

The ERM-certified PM material (ERM-CZ100 and PM_10_-like); dimethyl sulfoxide (DMSO); azo dye-impregnated collagen; fluorescent probe 2′; 7′-dichlorodihydroflurescin diacetate (DCFH-DA); 3-(4-5-dimethyl-2yl)-2-5-diphynyltetrasolium bromide (MTT); and MMP-1, 2, 8, 9, and 13 ELISA kits were purchased from Sigma-Aldrich (St. Louis, MO, USA). The phosphate-buffered saline (PBS, 1X), Dulbecco’s modified Eagle medium (DMEM), Ham’s Nutrient Mixtures medium (F-12), fetal bovine serum (FBS), and penicillin/streptomycin (P/S) were purchased from Gibco BRL (Life Technologies, Burlington, ON, Canada). Antibodies against NF-κB p50 and p65; phosphorylated AP-1 (p-c-Jun); nucleolin; MAPKs including p-ERK, p-JNK, and p-p38; and GAPDH were purchased from Santa Cruz Biotechnology (Santa Cruz, CA, USA). Anti-mouse and anti-rabbit IgG antibodies were purchased from Cell Signaling Technology (Beverly, MA, USA). PIP EIA kit was purchased from TaKaRa Bio Inc. (Shiga, Japan). All other chemicals used in this study were analytical grade.

### 4.2. Preparation of DPHC

The phenolic compound, DPHC, was obtained from *I. okamurae*. The separation and identification methods were described in the previous study [42]. Briefly, *I. okamurae* was extracted with aqueous ethanol (50%, v/v), and the ethanol extract of *I. okamurae* (IOEE) was obtained. IOEE was fractionated by a centrifugal partition chromatography (Tokyo, Japan) with a two-phase solvent system, which was composed of n-hexane: ethyl acetate: methanol: water (1:9:4.5:6.5, v/v). DPHC was further purified by a high-performance liquid chromatography (Waters, Mailford, USA) and identified by a mass spectrometer (Bruker Daltonics, Breman, Germany). DPHC stock solution (100 mM, DMSO) was diluted to different concentrations (0.5 mM, 1 mM, and 2 mM) with 1X PBS for experiments.

### 4.3. Cell Culture

HDF cells (ATCC^®^ PCS20101™) were purchased from ATCC (American Type Culture Collection, Manassas, VA, USA). Cells (passage 8) were cultured in the medium (DMEM and F-12, 3:1) and supplemented with 1% P/S and 10% heat-inactivated FBS. Cells were sub-cultured every 5 days and seeded at a concentration of 5 × 10^4^ cells/mL for experiments.

### 4.4. Determination of the Effect of DPHC on PM-Induced Cytotoxicity in HDF Cells

The effect of DPHC on ERM-CZ-100-induced cytotoxicity in HDF cells was determined by evaluating the intracellular ROS generation and cell viability. HDF cells were seeded in a 24-well plate and incubated for 24 h. Cells were treated with DPHC (25, 50, and 100 μM) for 1h, and ERM-CZ-100 (200 μg/mL) was added into each well. After 3 h, the intracellular ROS levels of ERM-CZ-100-treated HDF cells were evaluated by DCF-DA assay following the protocol described previously [43,44]. After 24 h, the viabilities of ERM-CZ-100-treated HDF cells were measured by MTT assay according to the methods described previously [45,46].

### 4.5. Determination of the Effect of DPHC on Collagen Synthesis, MMP Expression, and Intracellular Collagenase Activity

HDF cells were seeded and incubated for 24 h. Cells were treated with DPHC (25, 50, and 100 μM) for 1 h and then stimulated with ERM-CZ-100 (200 μg/mL). After 24 h incubation, the cell culture media was collected for analysis collagen content and MMPs levels using the commercial ELISA kits based on the manufacturer’s instructions. The cells were harvested for evaluation of the relative intracellular collagenase activity following the protocol described in the previous study [8].

### 4.6. Determination of the Effect of DPHC on NF-κB, AP-1, and MAPKs in PM-Stimulated HDF Cells

The effect of DPHC on NF-κB, AP-1, and MAPKs in ERM-CZ-100-stimulated HDF cells was assessed by Western blot assay. HDF cells were seeded and treated with DPHC for 1 h. The DPHC-treated cells were stimulated with ERM-CZ-100 for 1 h, and then the cells were harvested to investigate activated MAPKs levels. The DPHC-treated cells were stimulated with ERM-CZ-100 for 6 h, and then the cells were harvested to investigate activated NF-κB and AP-1 levels. The Western blot analysis were performed as described previously [8].
